# Atherosclerosis increases adventitial pressure and limits solute transport via fluid-balance mechanisms

**DOI:** 10.1007/s10237-025-02000-2

**Published:** 2025-09-01

**Authors:** Willy V. Bonneuil, Daniel J. Watson, Sarajo K. Mohanta, Andreas J. R. Habenicht, James E. Moore, Jennifer Frattolin

**Affiliations:** 1 Department of Bioengineering, Imperial College London, London, United Kingdom; 2 Institut de Physique de Rennes, UMR 6251, Université de Rennes, Rennes, France; 3 Institute for Cardiovascular Prevention, Ludwig-Maximilians-University, Munich, Germany; 4 Department of Cardiology, Nanjing Drum Tower Hospital of Nanjing University Medical School, Nanjing, Jiangsu China; 5 School of Engineering, University of British Columbia, Kelowna, BC Canada

**Keywords:** Atherosclerosis, Adventitia, Microvasculature, Fluid transport, Solute transport

## Abstract

The adventitia of blood vessels is their structural interface with surrounding tissues and may also contribute importantly to atherogenesis. Adventitial vasa vasorum and lymphatic vessels provide sources and sinks of interstitial fluid and solutes and remodel in disease. We constructed a mathematical model to investigate how soluble disease mediators, including lipoproteins and cytokines, are transported through the artery wall in healthy and atherosclerotic conditions. We derived model parameters from *in vivo* measurements where possible and extensively investigated the sensitivity of fluid flow and solute transport to them. Adventitial interstitial fluid pressure is predicted to increase in atherosclerosis because of a shift in transmural fluxes across vasa vasorum and lymphatics. In healthy conditions, 40–80% of the fluid gathered by lymphatics originates from vasa vasorum, and this increases to 60–90% in atherosclerosis. The increased dilution of fluid flowing from the inner layers in atherosclerosis implies that solute transport from the media to the adventitia is impaired. This implies increased concentration gradients near the external elastic lamina that may increase immune-cell retention there, and decreased gradients in the outer adventitia that may reduce immune-cell attraction from there.

## Introduction

**Fig. 1:**
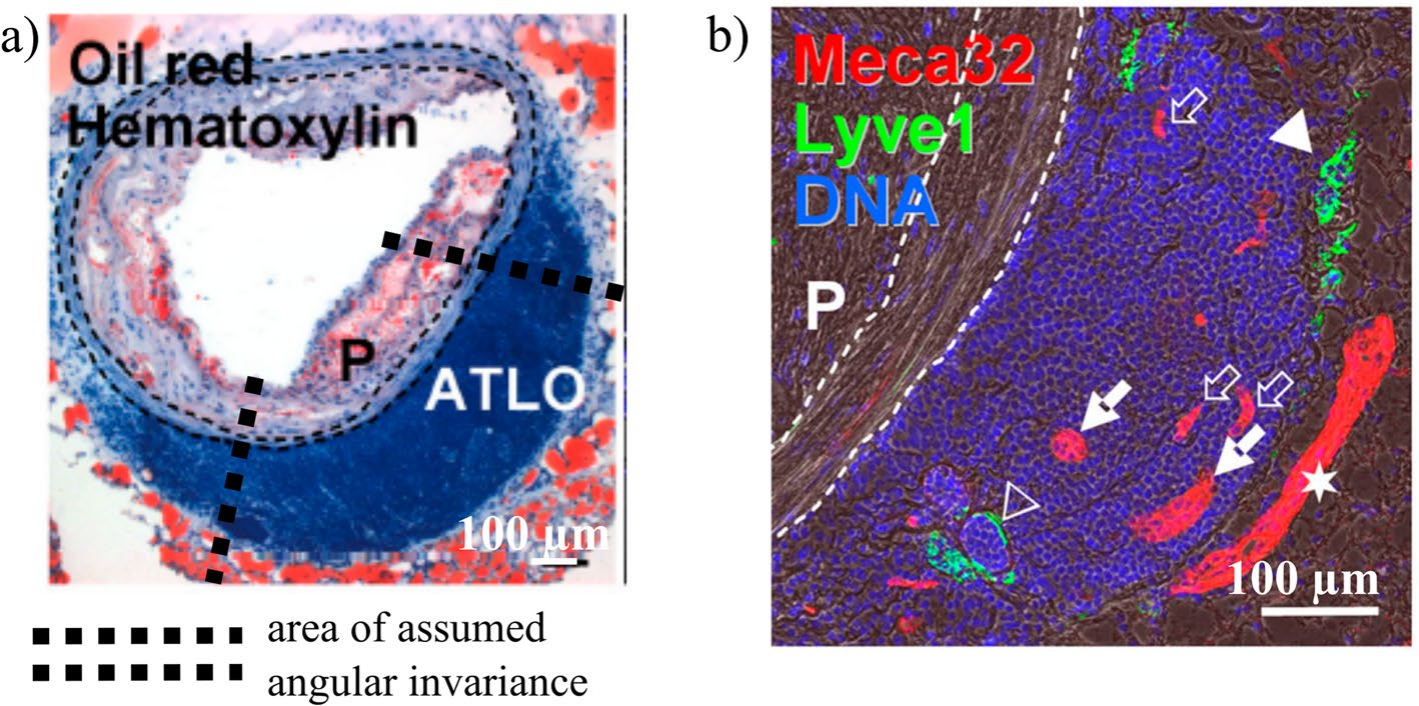
Histological section of murine atherosclerotic abdominal aorta with ATLO, representative of the domain used in this model and chosen for its macroscopic anatomical clarity. Cell nuclei stained in blue (haematoxylin), indicating the dense cellularity of the ATLO. P: plaque. Scale bar: 100 µm. From (Grabner et al (2009), available under a CC-BY-NC-SA 3.0 license. Immunostained section of ATLO for microvessels and cell nuclei (Meca32 for endothelial cells, LYVE-1 for lymphatic endothelial cells, DAPI for DNA of cell nuclei, P: plaque). Open arrows: capillary vasa vasorum, filled arrows: HEV, open triangles: abnormal distended lymphatic vessels, closed triangles: normal lymphatic vessels, asterisk: vena cava. Stained cells in the ATLO include resident cells in the adventitia (e.g. fibroblasts) and immune cells. From Grabner et al (2009), available under a CC BY-NC-SA 3.0 license

The development of atherosclerosis is accompanied by inflammation in the adventitia, which involves several changes in cellular population and tissue characteristics (Michel et al (2007); Campbell et al (2012); Tinajero and Gotlieb (2020)). In particular, the adventitia is the primary site of immune-cell accumulation, with up to two orders of magnitude more immune cells than in the plaque (Moos et al (2005)). This accumulation is also a feature of other types of cardiovascular disease, including aneurysms, and can present various degrees of structural organisation. Initial lymphocyte aggregates progressively form separate B and T cell zones, then tertiary lymphoid organs which participate in the control of the immune response associated with atherosclerosis (Hu et al (2015); Yin et al (2016)). Adventitial tertiary lymphoid organs (ATLO) extend from the external elastic lamina (EEL) into the adventitial space (Fig. 1a) and are crossed by vasa vasorum, including high endothelial venules (HEV), and lymphatic vessels (Fig. 1b). The complexity of adventitial immune structures was shown to correlate with the severity of disease on the luminal side of the affected vessel. ATLOs were observed in abdominal aortas in mice (Grabner et al (2009)) and at various locations in humans, including abdominal aortas (Guedj et al (2014); Dutertre et al (2014)) and coronary arteries (Akhavanpoor et al (2018)), where they were observed to correlate with lesion size and plaque vulnerability.

The structural changes occurring from the healthy state suggest that these pathological developments may be related to fluid movement and solute transport. In the media, atherosclerosis induces a reduction in thickness (Waller et al (1992)) and in resistance to fluid flow due to elastin degradation (Cocciolone et al (2018); Guang et al (2021a)). Vasa vasorum become more permeable and more prevalent (Ritman and Lerman (2007); Rademakers et al (2013); Philippi (2022)). Lymphatic vessels were reported to be denser by many (Kutkut et al (2015); Rademakers et al (2017); Drosos et al (2019); Yeo et al (2020)), but not all (Taher et al (2016)), studies. Their drainage rate is difficult to measure, but a recent study observed that solute transport from the adventitia to lymph nodes was delayed (Yeo et al (2020)). Lymphatic vessels are a sink for arterial cholesterol (Martel et al (2013)), and the role of their function in disease development and outcome is increasingly recognised (Feng et al (2022); Yeo et al (2021)). Their function as solute sinks implies that they play a key role in shaping concentration gradients. That may specifically affect gradients of chemokines produced by vascular smooth muscle cells (VSMC) and adventitial stromal cells. Those gradients are essential to immune-cell attraction and retention and in ATLO organisation (Grabner et al (2009); Guedj et al (2014)).

Detailed fluid flow and solute transport modelling studies of the arterial wall have focused on the portion of the wall between the lumen and the EEL. For instance, the explicit inclusion of the pores of the internal elastic lamina was shown to significantly modify the shear stresses and low-density lipoprotein (LDL) concentrations sensed by the VSMCs that are near the pores (Tada and Tarbell (2000, 2004)). The macromolecules albumin and LDL are key contributors to tissue homeostasis and to the intimal aspect of atherosclerosis, respectively, and their transport from the artery lumen into the intima and media has been well studied (Karner et al (2001); Tarbell (2003); Tada and Tarbell (2004); Ai and Vafai (2006); Chung and Vafai (2013)). The adventitia is usually included as a pressure boundary condition in fluid flow studies (30 mmHg (Ai and Vafai (2006)) or 40 mmHg (Karner et al (2001))) even though, to the best of our knowledge, no measurements of the pressure at the EEL have been reported. Interstitial pressure has been measured in various tissues at values often in the low units of mmHg (positive or negative) (Guyton et al (1971); Brace (1981)). One could estimate the pressure at the EEL to be close to this value but slightly higher because of adventitial resistance on the transwall flow pathway. In simulations of solute transport from the lumen into the wall, the adventitia is commonly included as a zero-flux boundary (Ai and Vafai (2006)). The increasingly recognised importance of the adventitia and perivascular adipose tissue (PVAT) (Brown et al (2014); Qi et al (2018); Hillock-Watling and Gotlieb (2022)) in atherogenesis suggests that there may be important mass transport phenomena across the tunica media. Fluid flow and transport modelling could contribute to quantifying how mediators of lesion status are transported from the inner to the outer layers and how they affect the formation of ATLOs.

The objective of this study is to estimate fluid flow conditions in the outer layers of a coronary arterial wall and thereby to identify fluid and solute transport patterns that may play a role in disease progression. Models of solute transport with advection, diffusion, and reaction have already shed light on the essential role of advection in shaping solute gradients, including of chemokines (Fleury et al (2006); Jafarnejad et al (2017)), and they have the potential to produce similar insights for the peripheral artery wall layers. To achieve that, we developed a model of fluid flow and solute transport in an artery wall comprising all compartments from the inner layers to the PVAT, vasa vasorum, and lymphatics.

## Methods

### Radial fluid flow model

**Fig. 2:**
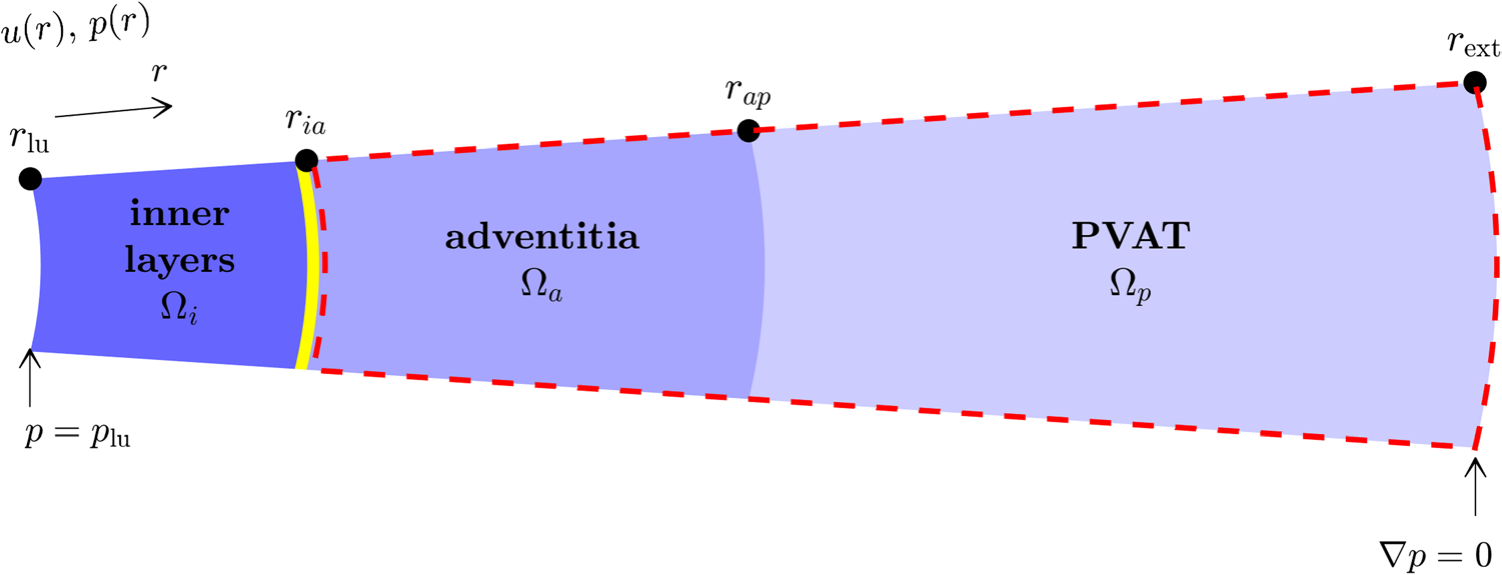
Computational domain based on the histological cross section in Fig. 1a. Fluid velocity and pressure assumed to only depend on the distance *r* from the lumen centre. Yellow band: EEL. Area bordered by dashed red line: mass exchange with vasa vasorum and lymphatics

Fluid flow is modelled in healthy and atherosclerotic configurations. A cylindrical section of arterial wall extending radially from the endothelium to the outer limit of the PVAT is assumed to be circumferentially invariant (Fig. 2). It is separated into three sequential porous domains: the inner layers (intima and media), the adventitia, and the PVAT. Those are, respectively, noted $\Omega _i$, $\Omega _a$, and $\Omega _p$. The tissue is assumed to be non-compliant and to resist fluid flow according to Darcy’s law:

1 $$\begin{aligned} u = -\frac{k(r)}{\mu }\frac{\textrm{d} p}{\textrm{d} r} \end{aligned}$$

The permeability *k* is a stepwise function that is constant in each domain:

2 $$\begin{aligned} k |_{\Omega _{\alpha }} = k_{\alpha }, \end{aligned}$$

and the domains are linked by continuity of pressure and velocity at their interfaces.

Tissue irrigation from vasa vasorum and drainage by adventitial and perivascular lymphatics are included in the adventitia and PVAT. Vasa vasorum irrigation follows the revised Starling hypothesis (Michel (1997); Weinbaum et al (2021)). Under the revised hypothesis, the steady-state relationship between the driving trans-capillary pressure difference and the filtration flux $J_v$ may be described by a hyperbola of asymptotes equal to the original Starling principle at highly positive pressure differences and to zero at highly negative pressure differences. We thus approximate the revised principle as a piece-wise linear function of the trans-capillary pressure difference:

3 $$\begin{aligned} J_v&= {\left\{ \begin{array}{ll} l_{p,v}\,\bar{A}_v\,\delta P(p) & \text {if } \delta P > 0 \\ 0 & \text {otherwise} \end{array}\right. } \end{aligned}$$

4 $$\begin{aligned} \delta P(p)&= p_v-p-\sigma (\pi -\pi _v) \end{aligned}$$

Vasa vasorum density is most often reported in number of vessels per surface area. To use such data, we approximate the surface area density of vasa vasorum $\bar{A}_v$ with the total circumference of vasa vasorum per unit cross-sectional area $N_v\pi d_v$, with $d_v$ a capillary diameter of 10 µm.

Lymphatic drainage is included as a flow rate outlet, assumed uniformly distributed in the tissue. It is also assumed proportional to its flux across a single primary valve, which was modelled by Heppell et al. (Heppell et al (2015)) as a flux per unit length of vessel and noted $\tilde{q}_{\ell }$. The lymphatic flux is then

5 $$\begin{aligned} J_{\ell } = - N_{\ell }\,\tilde{q}_{\ell }\,\bar{l}_{\ell } \end{aligned}$$

with $N_{\ell }$ the number of vessels per surface area and $\bar{l}_{\ell }$ the average length of lymphatic vessel per unit radial thickness, which is non-restrictively set to 1 here. The resulting mass conservation equation is:

6 $$\begin{aligned} \frac{1}{r}\frac{\textrm{d} }{\textrm{d} r} \left( r u \right) = J_v + J_{\ell } \end{aligned}$$

We assume that all fluid entering the arterial periphery between the lumen and the outer boundary of the PVAT is drained by lymphatic vessels in the arterial periphery. To close the system, we reciprocally assume that those lymphatics only drain fluid originating from the arterial lumen or the vasa vasorum. We thus impose a no-flow boundary condition at the outer PVAT, enforced by the pressure gradient:

7 $$\begin{aligned} \frac{\textrm{d} p}{\textrm{d} r} |_{r_{\textrm{ext}}} = 0 \end{aligned}$$

A steady pressure is imposed at the artery lumen:

8 $$\begin{aligned} p(0) = p_\textrm{lu} \end{aligned}$$

The atherosclerotic configuration presents thicker inner layers and a thicker adventitia (Gradus-Pizlo et al (2003)), a higher inner-layer permeability (Baldwin et al (1997)), denser and more permeable vasa vasorum (Kwon et al (1998); Rademakers et al (2013)), and denser and slower-draining lymphatics (Rademakers et al (2017); Drosos et al (2019); Yeo et al (2020)) (quantitative parameters in Supplementary Tables 1 and 2). The model is solved in Matlab (MathWorks, USA) using the finite-difference solver bvp4c.

### Model parameters and parameter refinement

Many of the model parameters have either not been measured *in situ* or been measured at other vascular sites. To reduce the resulting uncertainty, we first implement the model so as to refine parameter ranges. This is done using a condition of physiologic plausibility for interstitial fluid pressure at the outer adventitia ($p_\textrm{ap}$). That pressure is expected to be between -10 and 5 mmHg, which covers the other tissues where it has been measured (Guyton et al (1971); Brace (1981)). To estimate whether a parameter set corresponds to that interval, we consider a scalar approximation of mass exchange (Supplementary Information) where it occurs in the PVAT only and at a constant pressure, noted $p_\textrm{const}$. Under that assumption, the whole tissue ratio of outgoing to incoming mass fluxes,

9 $$\begin{aligned} \frac{J_\textrm{out}}{J_\textrm{in}}&= \frac{J_{\ell }}{J_i+J_v} \nonumber \\&= \frac{N_{\ell }\,\tilde{q}_{\ell }}{\dfrac{\ln {(r_{ap}/r_\textrm{lu})}k_i k_a}{\ln {(r_{ap}/r_{ia})}k_i+\ln {(r_{ia}/r_\textrm{lu})}k_a}\dfrac{2(p_\textrm{lu}-p_\textrm{const})}{\mu (t_p^2+2r_\textrm{ap}t_p)}+l_{p,v}\,\bar{A}_v\,\delta P(p_\textrm{const})} \end{aligned}$$

is approximately 1. The left-hand term of the denominator expresses Darcy’s law across the inner layers and adventitia. The right-hand term expresses Starling’s law when the tissue hydrostatic pressure is $p_\textrm{const}$. Let now the mass-balance deviation $\Lambda$ be defined as the ratio of outgoing to incoming mass fluxes if all mass exchange occurs in the PVAT at a constant, arbitrary, pressure $p_\textrm{test}$.

10 $$\begin{aligned} \Lambda = \frac{N_{\ell }\,\tilde{q}_{\ell }}{\dfrac{\ln {(r_{ap}/r_\textrm{lu})}k_i k_a}{\ln {(r_{ap}/r_{ia})}k_i+\ln {(r_{ia}/r_\textrm{lu})}k_a}\dfrac{2(p_\textrm{lu}-p_\textrm{test})}{\mu (t_p^2+2r_\textrm{ap}t_p)}+l_{p,v}\,\bar{A}_v\,\delta P(p_\textrm{test})} \end{aligned}$$

**Fig. 3:**
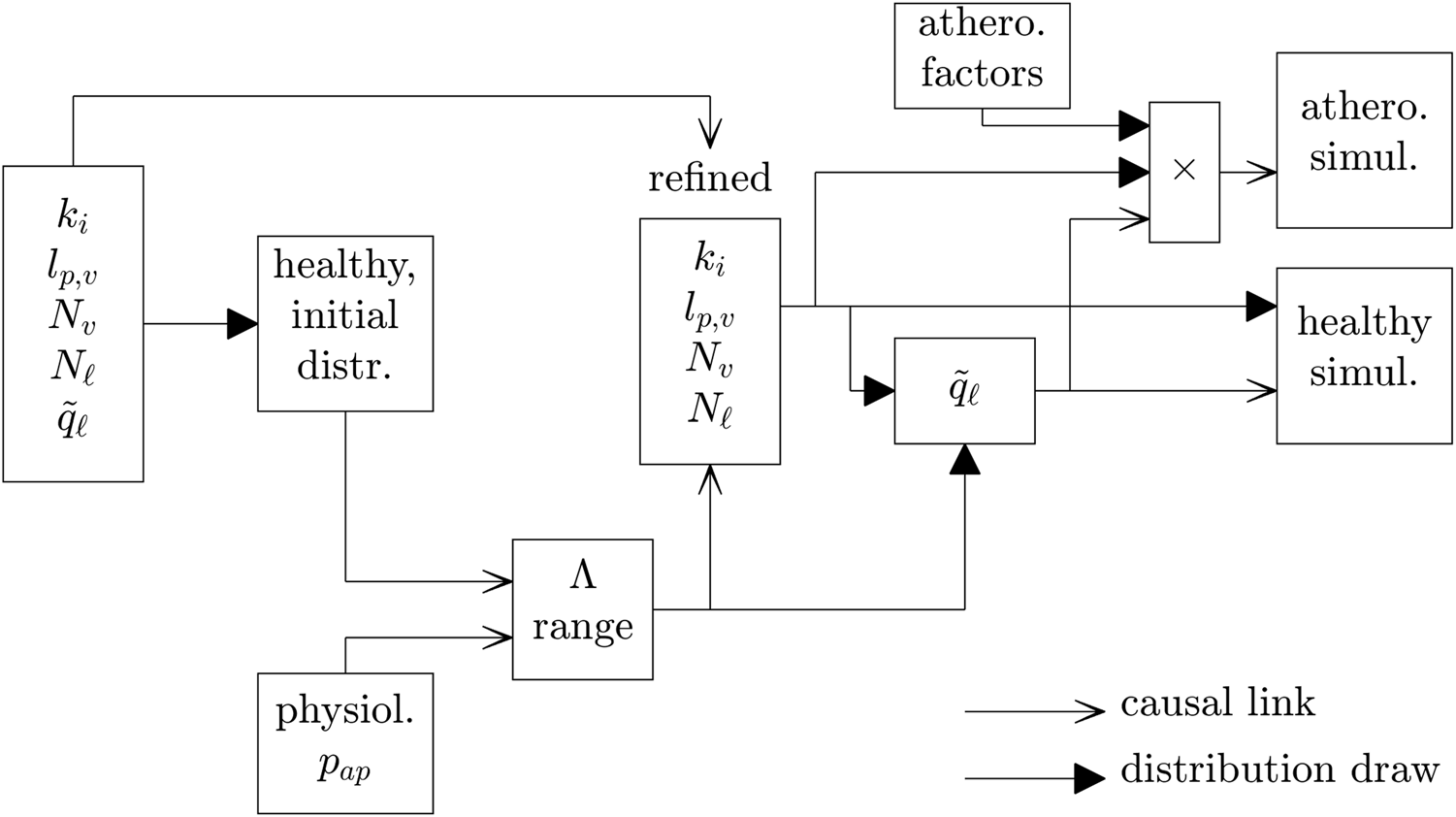
Workflow of parameter refinement based on the results of $10^4$ simulations with the initial parameter distributions in Supplementary Table 1. “distr.": distributions. “athero. factors": factors multiplying parameters drawn from healthy ranges (see Supplementary Table 2). “$\times$": multiplication. “simul.": simulations

If $p_\textrm{test} \approx p_\textrm{const}$, $p_\textrm{test}$ is a good approximation of tissue pressure and $\Lambda \approx 1$. If $p_\textrm{test} \gg p_\textrm{const}$, the denominator terms in $\Lambda$ are overestimations of the incoming mass fluxes and $\Lambda \ll 1$, and vice versa. We apply $p_\textrm{test} = -2.5$ mmHg (the mean of the physiologic interval) onto $10^4$ simulations in the healthy configuration to establish the physiologic interval of $\Lambda$. That interval is incorporated into subsequent simulations by drawing $\Lambda$ from its physiologic interval and all other parameters in their range refined by the $\Lambda$ condition, except $\tilde{q}_{\ell }$. The lymphatic drainage rate is the parameter for which literature estimations are most uncertain, so we specify it to satisfy physiologic outer adventitial pressure before solving the model (Fig. 3).

The parameter distributions used in the initial set of simulations and those for atherosclerosis-induced changes are taken from the literature or our assumptions (Supplementary Tables 1 and 2). They are drawn from Gaussian distributions when literature sources specified mean and standard deviation and from uniform or log-uniform distributions when the literature provides no basis for their estimation.

The sensitivity of the mass-balance deviation $\Lambda$ is estimated at the baseline case (means of the parameter distributions in Supplementary Table 1) at the first order by comparing the derivatives of $\Lambda$ with respect to each of the three mass flow rates. The sensitivity of the lumen–wall flow rate is similarly estimated by comparing its derivatives with respect to $\sqrt{k_i}$, $\sqrt{k_a}$ and $t_p$.

### Solute transport model

The transport of non-binding solutes penetrating the periphery from the lumen or secreted in the inner layers is modelled by a steady-state advection–diffusion equation with microvascular sink fluxes $\varphi _v$ into vasa vasorum and $\varphi _{\ell }$ into lymphatic vessels. Solute concentration is noted *c*, *D* is the diffusivity in tissue that we assume uniform, and $\kappa _v$ is the diffusivity across the vasa vasorum endothelium:

11a $$\begin{aligned} \frac{D}{r}\frac{\textrm{d} }{\textrm{d} r} \left( r \frac{\textrm{d} c}{\textrm{d} r} \right) - \frac{\textrm{d} }{\textrm{d} r} (uc) + \varphi _v + \varphi _{\ell }&= 0 \end{aligned}$$

11b $$\begin{aligned} \varphi _v&= - (J_v + \kappa _v\,\bar{A}_v)\,c \end{aligned}$$

11c $$\begin{aligned} \varphi _{\ell }&= J_{\ell }\,c \end{aligned}$$

A concentration of $c_0$ is imposed either at the lumen ($r_\textrm{lu}$) or at the EEL ($r_{ia}$), and a diffusive flux of zero is imposed at one PVAT thickness away from the outer PVAT (where radial fluid flow is set to zero):

12 $$\begin{aligned} \frac{\textrm{d} c}{\textrm{d} r} |_{r_\textrm{ext}+t_p} = 0 \end{aligned}$$

This accounts for the fact that solutes continue *a priori* to diffuse beyond the distance where all fluid originating from the arterial lumen is drained. Tissue permeabilities and microvascular parameters are taken from their $\Lambda$-refined ranges and $\tilde{q}_{\ell }$ obtained from $\Lambda$, like in the fluid flow simulations.

Equation (11a) is non-dimensionalised with the following scales: $r = r^* (t_a+t_p)$; $u = u^* (k_\textrm{i}/\mu )(p_\textrm{lu}/t_i)$; $p = p^* p_\textrm{ref}$ where $p_\textrm{ref}$ = 1 mmHg, for hydrostatic and osmotic pressures; $c = c^* c_0$. This yields, omitting the asterisks:

13 $$\begin{aligned} \frac{1}{r}\frac{\textrm{d} }{\textrm{d} r} \left( r \frac{\textrm{d} c}{\textrm{d} r} \right) - \textrm{Pe}\,\frac{\textrm{d} }{\textrm{d} r} (uc) - \textrm{Da}_v\frac{\delta P(p)}{\delta P(0)} c - \textrm{R}_\textrm{d}c - \textrm{Da}_{\ell }c = 0 \end{aligned}$$

where the non-dimensional numbers $\mathrm Pe$ (Péclet), $\textrm{Da}_v$ (vasa vasorum Damköhler), $\textrm{Da}_{\ell }$ (lymphatic Damköhler), and $\textrm{R}_\textrm{d}$ (ratio of diffusive flux into vasa vasorum to diffusive flux in tissue) are defined as:

14a $$\begin{aligned} \mathrm Pe&= \frac{k_i}{\mu }\frac{p_\textrm{lu}}{t_i} (t_a+t_p) / D \end{aligned}$$

14b $$\begin{aligned} \textrm{Da}_v&= l_{p,v}\,\bar{A}_v\,\delta P(0) (t_a+t_p)^2/D \end{aligned}$$

14c $$\begin{aligned} \textrm{Da}_{\ell }&= N_{\ell }\,\tilde{q}_{\ell } (t_a+t_p)^2/D \end{aligned}$$

14d $$\begin{aligned} \textrm{R}_\textrm{d}&= \kappa _v\,\bar{A}_v\,(t_a+t_p)^2/D \end{aligned}$$

Solute transport is simulated for *D* over three orders of magnitude (100 µm$^2$/s to 1 µm$^2$/s). The upper value is approximately the diffusivity of chemokines (10 kDa) in water, the median value is approximately that of LDL (3 MDa), and the lower value represents, for example, LDL in a tissue where the effective diffusivity is 0.1. We are not aware of measurements of diffusivity in the adventitia or the PVAT, but studies of other tissues report effective diffusivities between 0.1 and 0.7 depending on molecular weight and charge (Casciari et al (1988); Syková and Nicholson (2008); Travascio et al (2020); Xia et al (2020); Guang et al (2021a, 2021b)). The [1,100] µm$^2$/s range therefore covers at least two solutes whose concentration in the arterial wall is key to atherosclerosis development and evolution. The diffusive flux ratio $\textrm{R}_\textrm{d}$ would be equal to 0.1 for albumin at the baseline vasa vasorum density (Supplementary Table 1) and the water diffusivity of albumin. Varying the effective tissue diffusivity between 0.1 and 1 and accounting for variations in endothelial permeability, whose dependence on molecular weight and charge is complex (Xia et al (2020)), we let $\textrm{R}_\textrm{d}$ vary between 0.01 and 10.

### Key model outputs

**Fig. 4:**
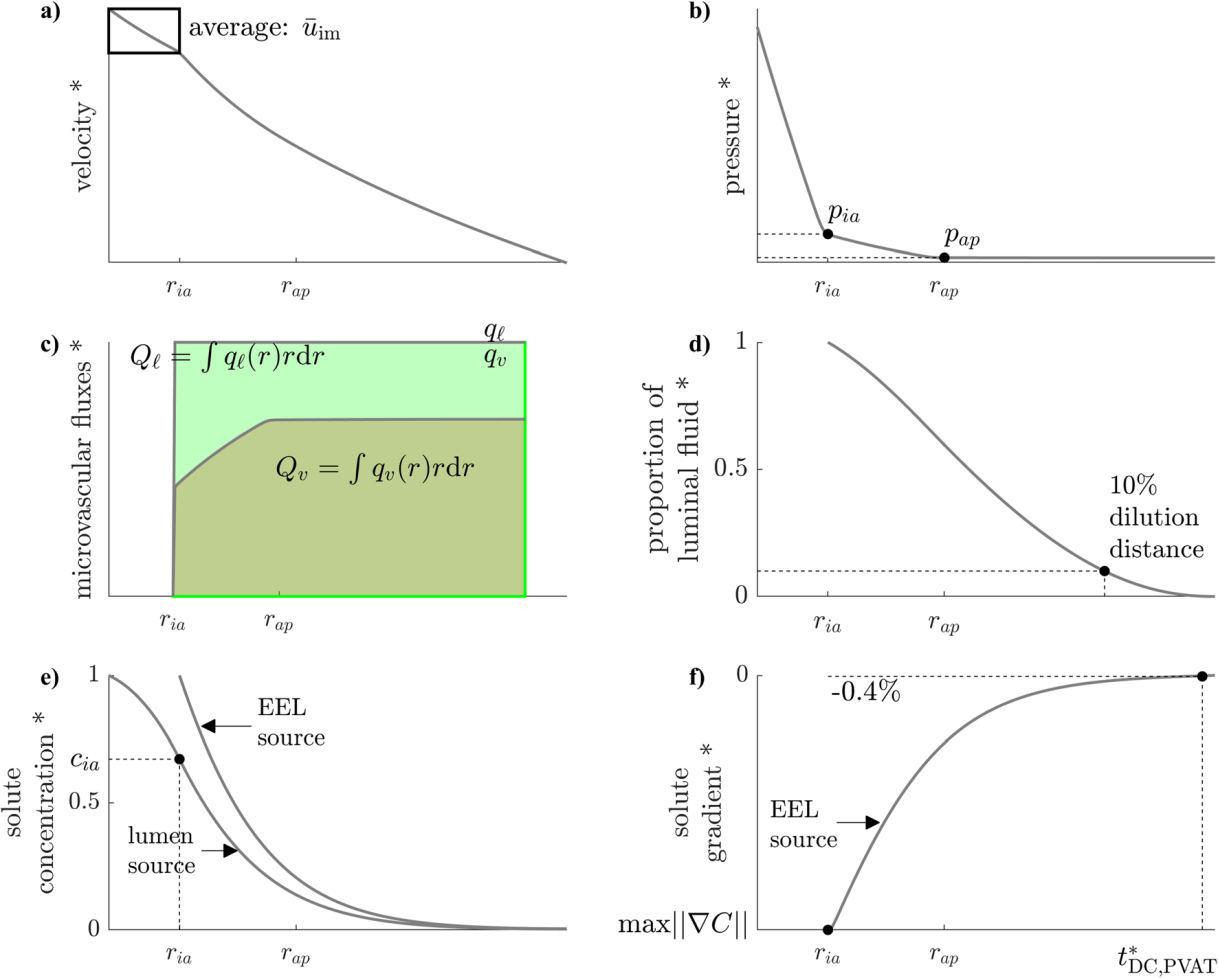
Model outputs on representative variable profiles. In axis labels, * = representative, *ia* = medial–adventitial boundary, *ap* = adventitial–PVAT boundary. **a** inner-layer average velocity. **b** medial–adventitial and outer adventitial pressures. **c** decomposition of total lymphatic outflow into fluid originating from the arterial lumen and fluid originating from vasa vasorum. **d** 10%-dilution distance. **e** concentration profile. **f** concentration gradient

The following fluid flow and solute transport quantities are analysed through their distributions obtained after parameter refinement:

- *Velocity halfway across the inner layers* (Fig. 4a): this can be compared to the multiple animal studies where wall conductivity was measured (Tedgui and Lever (1984); Baldwin et al (1992, 1997); Shou et al (2006); Nguyen et al (2015)). It is one of few quantities calculated by our whole-wall model that can be compared to experimental observations.
- *Medial–adventitial pressure* (Fig. 4b): this pressure was a boundary condition in previous fluid flow models of the intima and media (Karner et al (2001); Ai and Vafai (2006)), yet it has not been measured. Its value as estimated by our model could be used as a more-accurate boundary condition in future modelling studies of the inner layers.
- *Origin of lymph* (Fig. 4c): the ratio of vasa vasorum inflow to lymphatic outflow indicates how much the fluid flowing from the lumen across the inner layers is diluted. It thus gives an estimation of the concentration and gradient range of solutes for which convection dominates across the arterial wall (e.g. albumin (Baldwin et al (1997))). The vasa-to-lymph ratio is complemented by the dilution distance, i.e. the distance from the artery endothelium at which luminal fluid made up less than 10% of the interstitial fluid (Fig. 4d).
- *Concentration at the EEL* (Fig. 4e), noted $c_{ia}$: solutes that have their source in the lumen are subjected to two competing transport mechanisms in the wall: radially decreasing advection velocity favours their accumulation and microvascular sinks deplete them. The concentration at the EEL indicates how these two balance each other.
- *Maximum gradient in the adventitia* (Fig. 4f): for solutes with their source in the media, noted $\textrm{max} ||\nabla C||$. In the healthy configuration, it is an indicator of the intensity of chemotactic signals at the initial stages of immune-cell recruitment to the adventitia. In the atherosclerotic configuration, it indicates how strong those signals remain to sustain immune-cell retention and recruitment.
- *DC-CCL19 transport distance* (Fig. 4f): defined as the distance from the adventitia–PVAT border at which the gradient drops below 0.4 mm$^{-1}$. This is the threshold reported by Haessler et al (2011) for migration of dendritic cells (DC) in CCL19 gradients *in vitro*. That study is chosen as a reference because it has hitherto been the most precise in studying immune-cell migration as a function of chemotactic gradient value and because dendritic cells and CCL19 are both relevant to atherosclerosis. The ratio of that distance to PVAT thickness, noted $t^*_\textrm{DC,PVAT}$, is used to estimate the size of the region where gradients of chemokine produced by smooth muscle cells are strong enough to elicit directed immune-cell migration.

## Results

### Properties of peri-arterial fluid flow

**Fig. 5:**
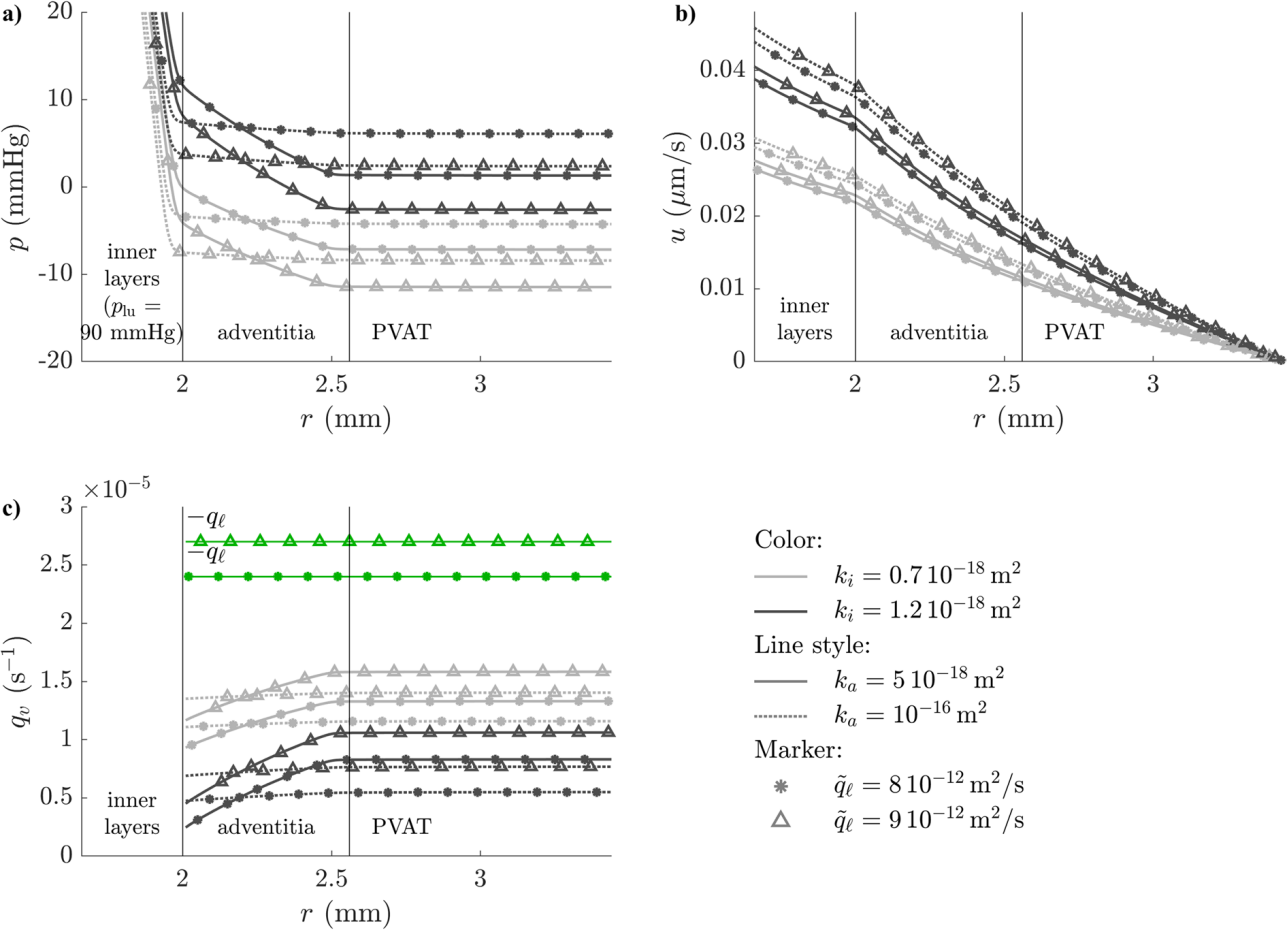
Selected profiles of fluid flow variables across the peri-arterial space (healthy configuration). Full parameters in Supplementary Table 3. **a**. Hydrostatic pressure. **b**. Interstitial fluid velocity. **c**. Microvascular fluxes, from vasa vasorum in shades of grey and into lymphatics in green

Selected cases from the healthy configuration with varying inner-layer permeability, adventitial permeability, and lymphatic drainage rate (all other parameters constant at the mean of their distribution) highlight the following fluid flow properties. Most of the pressure drop occurs across the inner layers, where permeability is lowest (Fig. 5a). The adventitial pressure drop varies with the ratio of adventitial to inner-layer permeability and was between 1 mmHg and 15 mmHg. The tissue pressure at the outer adventitia stabilises between -10 and 5 mmHg. Its value increases with lower lymphatic drainage and higher inner-layer and adventitial permeabilities. Interstitial fluid velocity continuously decreases from the lumen to the PVAT because of tissue widening and lymphatic drainage (Fig. 5b). Its value is largely determined by $k_i$ (higher for higher permeabilities). Mass fluxes from vasa vasorum, which are proportional to the trans-capillary pressure difference (Equation (4)), mirror interstitial pressure: they increase between the EEL and the PVAT and are higher for low $k_i$ and high $\tilde{q}_{\ell }$ (Fig. 5c). These configurations thus entail both more tissue oxygenation and more dilution of lumen-originating fluid. Lower adventitial permeabilities skew mass exchange with vasa vasorum towards the outer adventitia, thereby shifting the dilution of lumen-originating fluid outwards from the EEL.

### Peri-arterial pressure

#### Identification of physiologic range of mass-balance deviation

**Fig. 6:**
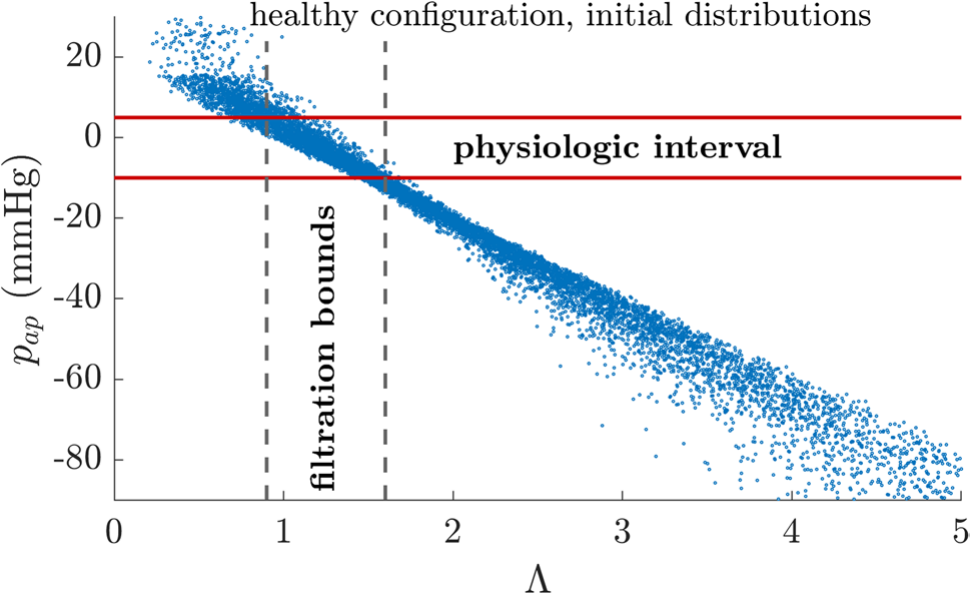
Filtration of parameter sets according to the physiologic plausibility of the outer adventitial pressure in the healthy configuration. The resulting condition on the mass-balance deviation is $\Lambda \in [0.9,1.6]$

The outer adventitial pressure $p_{ap}$ decreases linearly with the mass-balance deviation $\Lambda$ ($r^2 = 0.95$) in the initial simulation of the healthy configuration. A mass-balance deviation in [0.9,1.6] is required for $p_{ap}$ to be within the [-10,5] mmHg physiologic range (Fig. 6). This condition renders the microvascular parameters embedded in $\Lambda$ dependent on each other, but barely affects the individual distributions of vasa vasorum conductivity and density and only induces a slight shift to the left of the lymphatic-vessel density distribution (Supplementary Figure 1a–c). It yields a log-normal distribution, shifted to the left, of the lymphatic drainage rate (Supplementary Figure 1d). The sensitivities of $p_{ap}$ to each of the three mass flow rates are of the same order of magnitude (Supplementary Table 4), which indicates that none of the flow rates may be neglected.

#### Outer adventitial pressure elevated in atherosclerosis

**Fig. 7:**
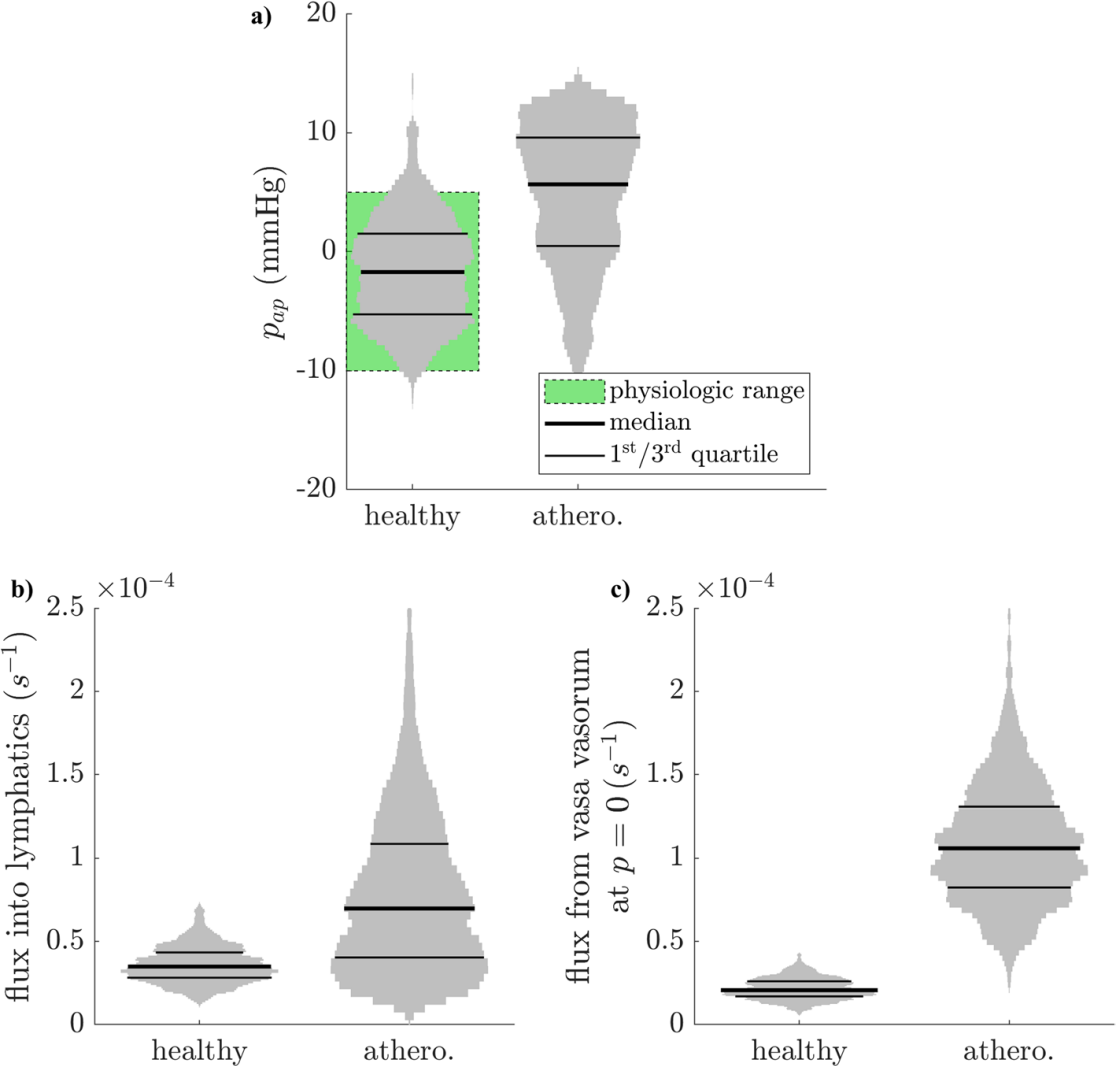
Outer adventitial pressure distribution in the healthy and atherosclerotic configurations, following parameter refinement by the mass-balance deviation. **a**. Mass flux into lymphatics. **b**. mass flux from vasa vasorum at zero hydrostatic tissue pressure

**Fig. 8:**
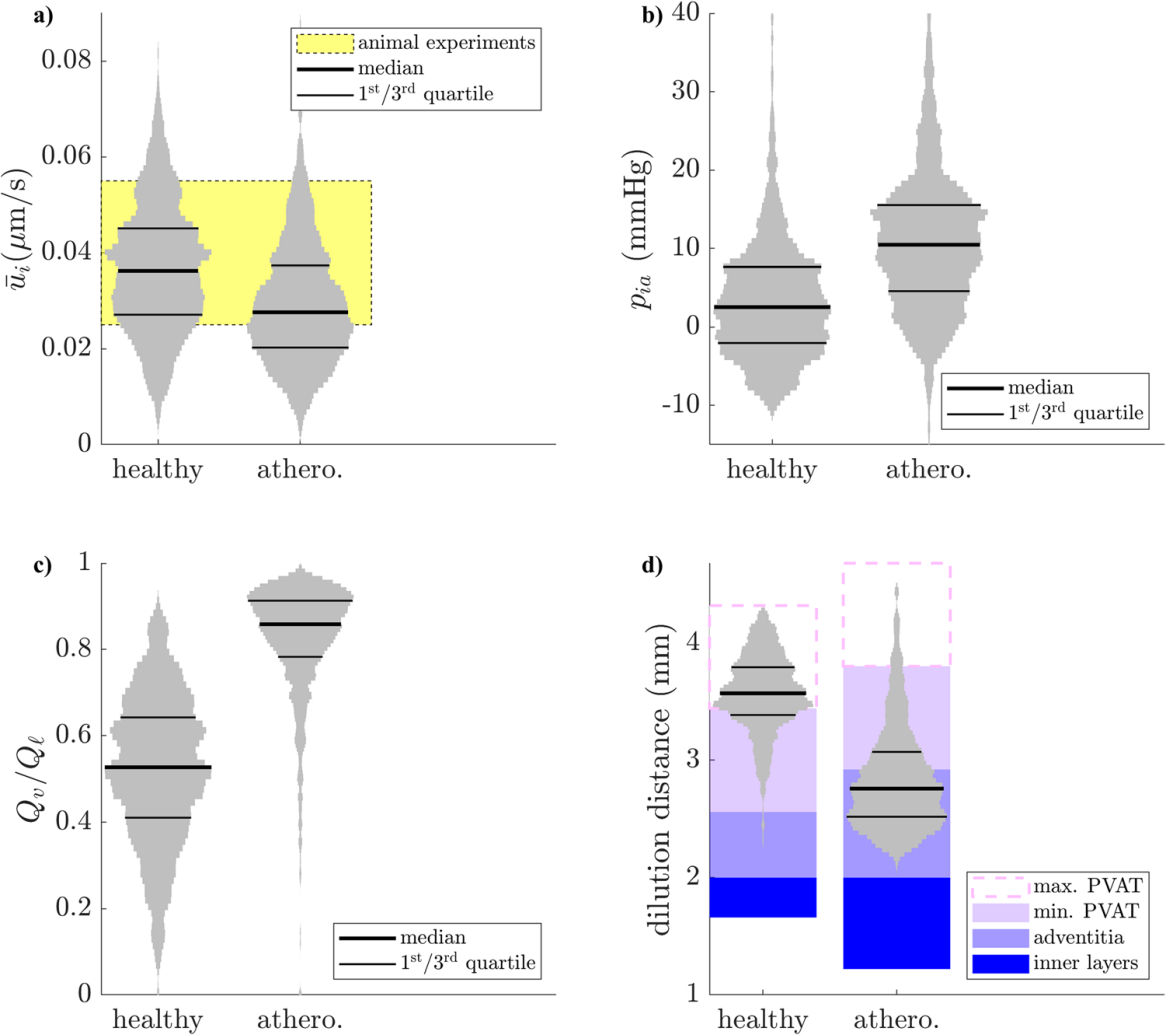
Key fluid flow outputs in healthy and atherosclerotic configuration, following parameter refinement by the mass-balance deviation. **a**. Inner-layer velocity. Reference animal experiments include (Tedgui and Lever (1984); Baldwin et al (1992, 1997); Shou et al (2006); Nguyen et al (2015)). **b**. Medial–adventitial pressure. **c**. Fraction of fluid originating from vasa vasorum in the wall-draining lymph. **d**. Dilution distance, i.e. distance from the centre of arterial lumen beyond which fluid of luminal origin makes up less than 10% of the interstitial fluid. Variation of PVAT thickness across cases represented by the white box with a dashed contour

The outer adventitial pressure remains within its physiologic plausibility interval after parameter refinement. It is between -10 and 5 mmHg in 90% of cases (Fig. 7a). It increases in the atherosclerotic configuration, taking positive values in 75% of cases and above 10 mmHg in 25% of cases. This increase can be explained by considering mass conservation for the whole tissue, which may be written as follows:

15 $$\begin{aligned} N_{\ell }\,\tilde{q}_{\ell }\,V = v_i\,\bar{A}_i + l_{p,v}\,\bar{A}_v\,\overline{\delta P}\,V \end{aligned}$$

where *V* is the volume of the peripheral layers, $\bar{A}_i$ is the average cross-sectional area of the inner layers, and $\overline{\delta P}$ is the average pressure driving vasa vasorum filtration (Equation (4)), weighted by tissue cross-sectional area. The tissue pressure increase thus obeys the following mechanism:

- lymphatic drainage lightly increases (from $3\,10^{-5}\,\textrm{s}^{-1}$ to $7\,10^{-5}\,\textrm{s}^{-1}$ on average, Fig. 7b), since the net effect of the reported increase in lymphatic density and decrease in drainage rate per lymphatic vessel is an increase in flow rate.
- mass conservation imposes a corresponding increase in the sum of flow rates into the tissue. Since the flow rate from the lumen is predicted to change only little with atherosclerosis (Fig. 8a), the flow rate from vasa vasorum to tissue increases, by an amount that matches the increase in total lymphatic outflow.
- since the vasa vasorum flow rate per unit pressure ($l_{p,v} N_v$) increases more than lymphatic drainage (Fig. 7c), mass conservation dictates that the driving pressure across the vasa vasorum endothelium must decrease. Since atherosclerosis does not affect the capillary hydrostatic pressure and the osmotic pressures, the hydrostatic tissue pressure increases.

### Conditions at the media–adventitia boundary

#### Inner-layer velocity reduced by a third in atherosclerosis

The inner-layer interstitial velocity is within the range of its measurements in animals in over 60% of healthy samples (0.025-$-$0.055 µm/s) and does not exceed that range by much (Fig. 8a). Its mean in the atherosclerotic simulations is 0.028 µm/s, which implies that solute convection across the inner layers, which is the dominant transport mechanism for, for example, albumin (Baldwin et al (1997)), is reduced by about a third in atherosclerosis. The sensitivity of the inner-layer mass flow rate is much higher to $\sqrt{k_i}$ than to $\sqrt{k_a}$ or $t_p$ (Supplementary Table 4), which indicates that inner-layer conductivity suffices to estimate the flow rate.

#### Media–adventitial pressure

The interstitial pressure at the media–adventitia boundary is predicted to be lower than the 30 mmHg assumed in inner-layer modelling studies. It was in the [-2,8] mmHg interval in 50% of healthy cases and slightly elevated in the atherosclerotic configuration, with 50% of simulations in the [5,17] mmHg interval (Fig. 8b).

### Origin of arterial wall-draining lymph

#### Most peri-arterial lymph is of microvascular origin

The majority of lymph draining the adventitia and PVAT is of vasa vasorum origin in most cases. In the healthy configuration, the proportion is at over 50% of lymph in 50% of cases (Fig. 8c). In the atherosclerotic configuration, this increases to over 75% of lymph in 75% of cases. This results from the increased total lymphatic outflow in atherosclerosis (Fig. 7b), whilst the flow rate across the arterial endothelium changes little (Fig. 8a), meaning that flow across the vasa vasorum walls increases alongside lymph outflow to satisfy mass balance.

#### High dilution of luminal fluid before PVAT in atherosclerosis

In the healthy configuration, fluid of arterial origin represents more than 10% of the interstitial fluid up until a large distance from the artery endothelium. The 10% dilution threshold is reached further than the adventitia in almost all cases (Fig. 8d). However, in the atherosclerotic configuration, the low proportion of fluid of luminal origin in the total lymph translates into a short dilution distance, shorter than the adventitial thickness in close to 70% of cases. This suggests that fluid in the outer adventitia and the PVAT is overwhelmingly of vasa vasorum origin in atherosclerosis. This increases the dilution of disease mediators being transported from the inner layers.

**Fig. 9:**
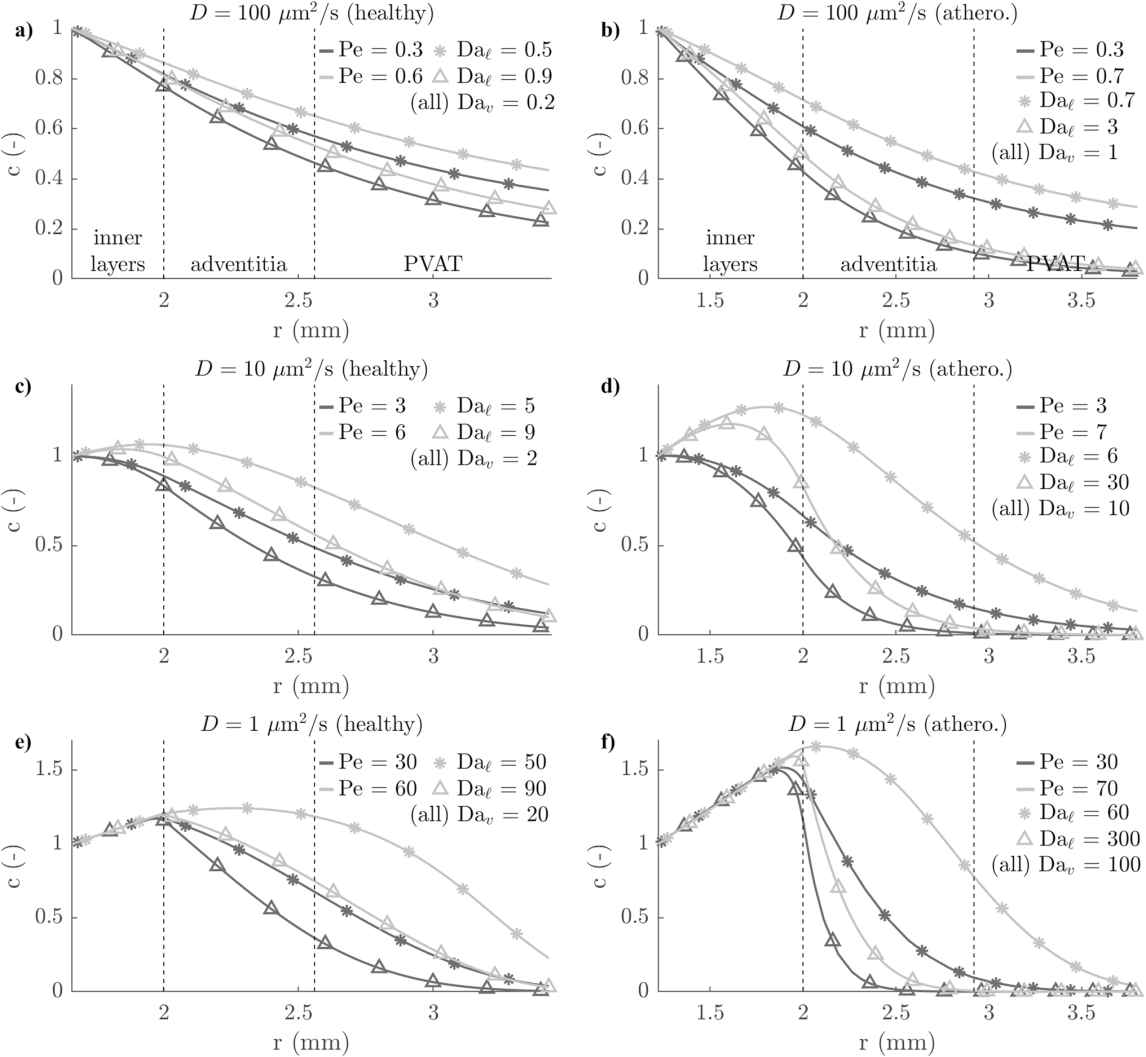
Selected profiles of solute concentration across the arterial wall. Grey curves: $k_i = 1.5\,10^{-18}$ m$^2$/s (healthy), $2.8\,10^{-18}$ m$^2$ (athero.). Black curves: $k_i = 0.6\,10^{-18}$ m$^2$/s (healthy), $1.2\,10^{-18}$ m$^2$ (athero.). Open triangles: $\tilde{q}_{\ell } = 1.5\,10^{-11}$ m$^2$/s (healthy), $10^{-11}$ m$^2$/s (athero.). Asterisks: $\tilde{q}_{\ell } = 0.8\,10^{-11}$ m$^2$/s (healthy), $0.2\,10^{-11}$ m$^2$/s (athero.) Full parameters of these cases in Supplementary Tables 5 (healthy cases) and 6 (atherosclerotic cases)

### Properties of solute transport across the arterial wall

Selected cases with the solute source in the lumen and varying inner-layer permeabilities, diffusivities in tissue, and lymphatic drainage rates (all other parameters constant at their baseline values) highlight the following transport properties. At $D = 100$ µm$^2$/s, adventitial microvascular sinks deplete solutes in all wall layers, over the entire range of interstitial convection velocities (Fig. 9a,b). Their higher magnitudes relative to diffusion in atherosclerosis reduce solute concentration in the adventitia and PVAT and increase the magnitude of the gradients near the source. At $D = 10$ µm$^2$/s, convection-dominated transport in the inner layers favours a small solute accumulation in the outer media because interstitial fluid slows down as it flows further away from the lumen (Fig. 9c). That accumulation appears for $\mathrm Pe\approx 5$, is greater with increasing $\mathrm Pe$, and shifts inward with increasing $\mathrm Da_{\ell }$. Thicker inner layers in atherosclerosis increase the magnitude of solute accumulation, and larger microvascular sinks lead to larger gradients in the outer media and inner adventitia and some concentration profiles dropping to essentially zero at the adventitia–PVAT boundary (Fig. 9d). At $D = 1$ µm$^2$/s, those effects are amplified (Fig. 9e,f). All concentration profiles display accumulation in the outer media, with solutes in atherosclerosis becoming 50% more concentrated close to the EEL. Some concentration profiles in atherosclerosis drop to essentially zero in the middle of the adventitia.

### Interstitial convection favours solute accumulation around the EEL

**Fig. 10:**
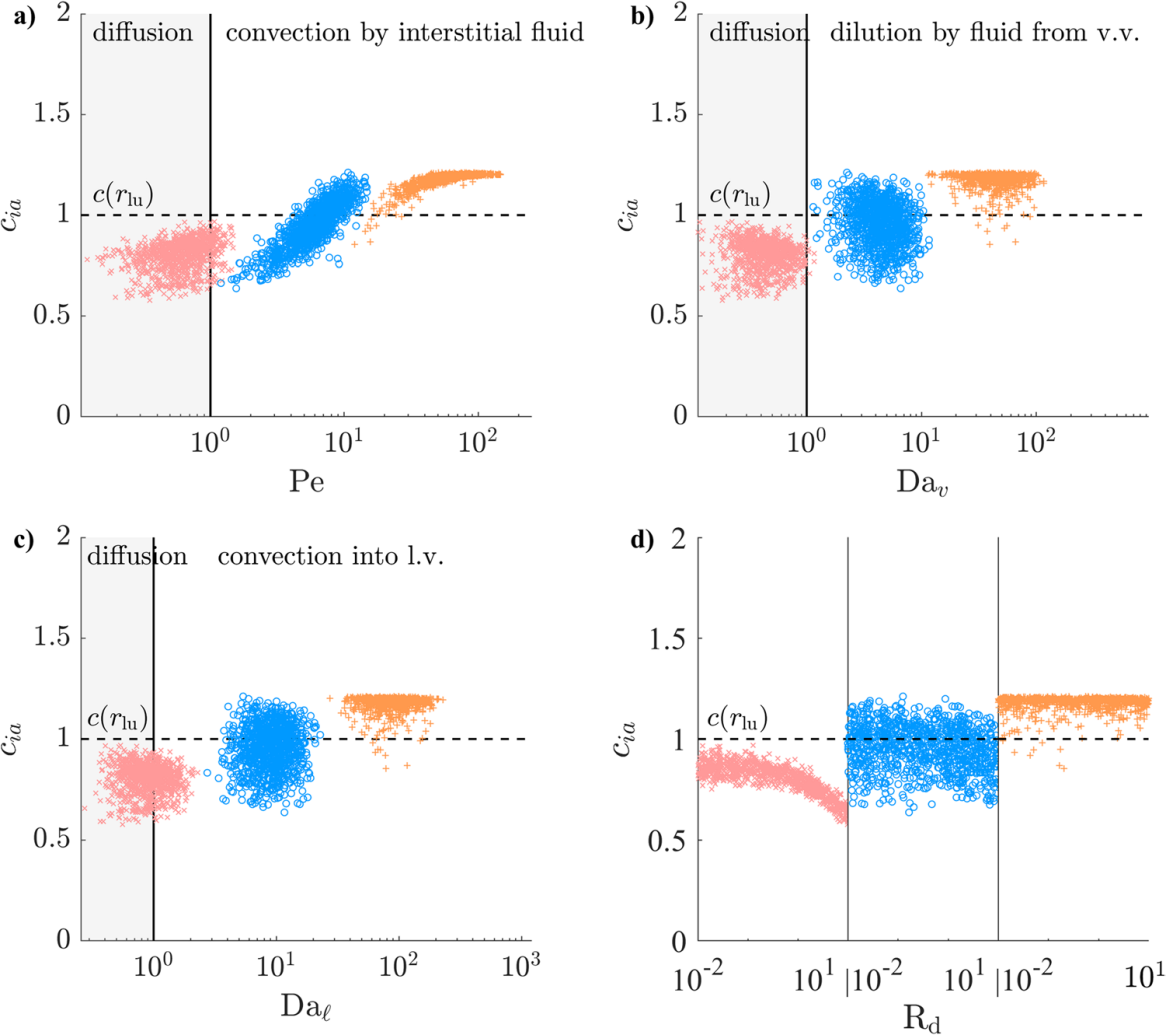
Solute concentration at the EEL (as defined in Fig. 4e) with solute source at the lumen (healthy configuration) as a function of the non-dimensional numbers governing Equation (11a). Regions of relative dominance of transport mechanisms separated by grey vertical line. ${\times }\; D=100$ µm$^2$/s, ${\circ }\; D=10$ m$^2$/s, ${+}\; D=1$ m$^2$/s

**Fig. 11:**
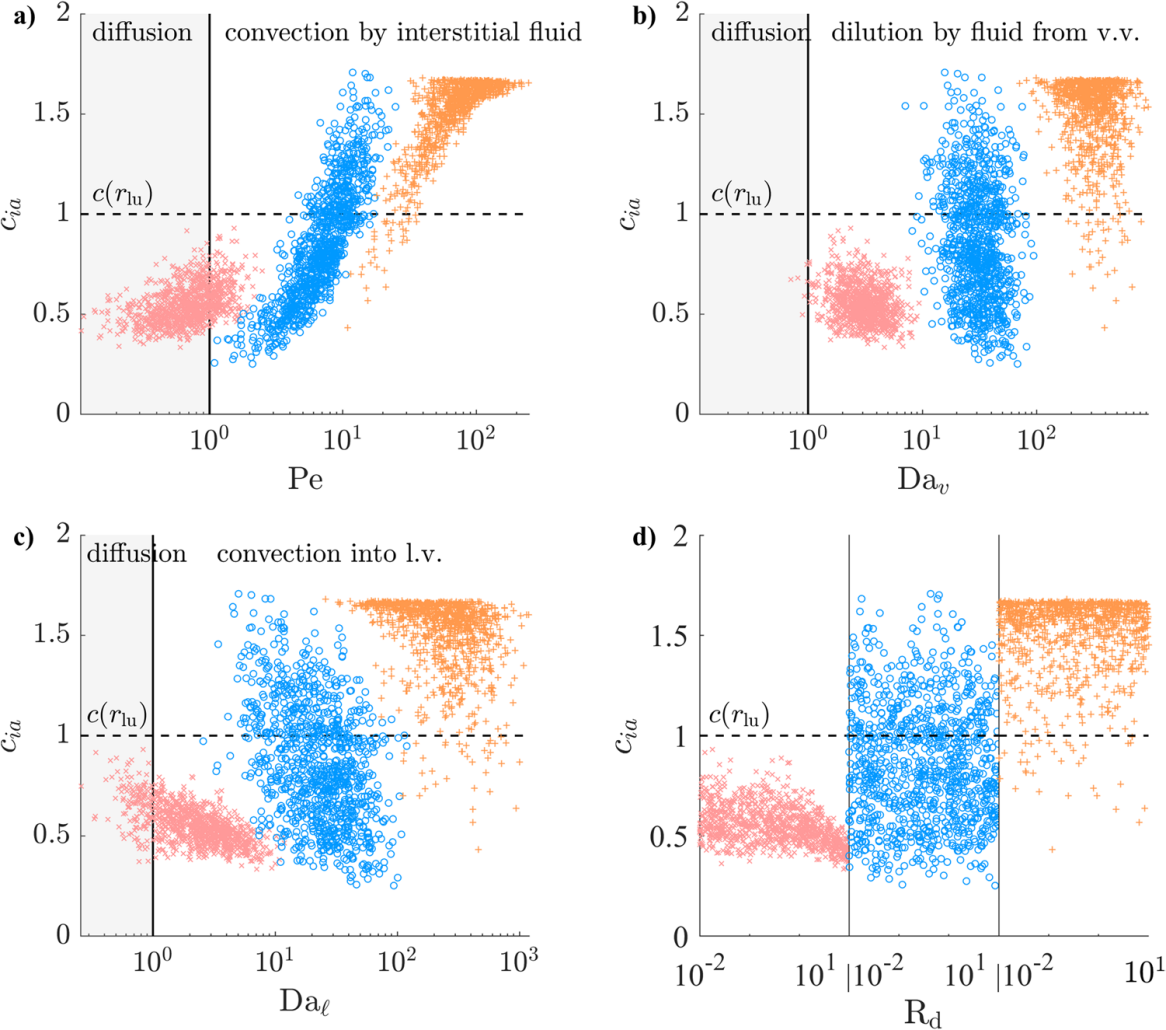
Solute concentration at the EEL (as defined in Fig. 4e) with solute source at the lumen (atherosclerotic configuration) as a function of the non-dimensional numbers governing Equation (11a). Regions of relative dominance of transport mechanisms separated by grey vertical line. ${\times }\; D=100$ µm$^2$/s, ${\circ }\; D=10$ m$^2$/s, ${+}\; D=1$ m$^2$/s

Solutes with a concentration source in the lumen display varying concentrations at the EEL (Figs. 10 and 11). At $D=100$ µm$^2$/s, the adventitial microvascular sinks ($\mathrm Da_v$, $\mathrm Da_{\ell }$ and $\textrm{R}_\textrm{d}$) all induce solute depletion and solute gradients are oriented towards the lumen in all simulated cases. At $D=10$ µm$^2$/s, interstitial convection dominates transport in the inner layers and favours solute accumulation because it diminishes with the distance from the lumen. The EEL concentration exhibits a strong correlation with $\mathrm Pe$ (Figs. 10a and 11a) and compensates the loss induced by the microvascular sinks when $\mathrm Pe>5$. In those cases, solute gradients are directed towards the EEL both in the inner layers and in the adventitia. At $D=1$ µm$^2$/s, this concerns nearly all simulated cases. In the healthy configuration, the EEL concentration varies between 0.6 and 1.2, but that range is much extended in atherosclerosis. Greater microvascular sink magnitudes enhance solute depletion, with combined high lymphatic drainage and low interstitial convection bringing $c_{ia}$ at $D=10$ µm$^2$/s down to 0.3 (Fig. 11a and c). Thicker inner layers enhance solute accumulation at equal $\mathrm Pe$, bringing $c_{ia}$ up to 1.7 when high interstitial convection is combined low lymphatic drainage.

### Maximal concentration gradient greatly increases in atherosclerosis

**Fig. 12:**
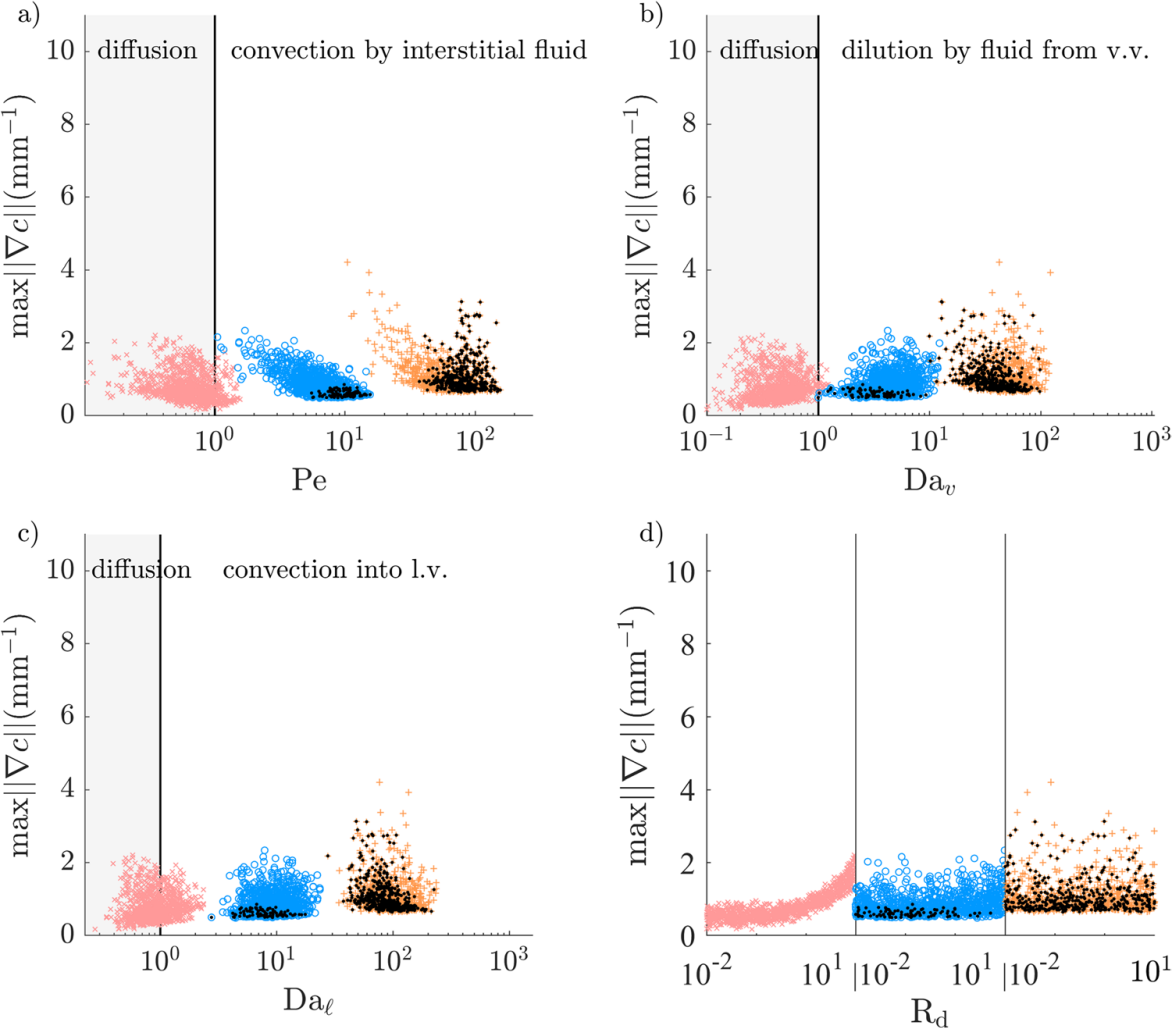
Maximum adventitial gradient (as defined in Fig. 4e) for solutes with an EEL source (healthy configuration), as a function of the non-dimensional numbers governing Equation (11a). Regions of relative dominance of transport mechanisms separated by grey vertical line. ${\times }\; D=100$ µm$^2$/s, ${\circ }\; D=10$ m$^2$/s, ${+}\; D=1$ m$^2$/s, ${\varvec{\cdot }}$ maximum concentration not at the EEL

For solutes with an EEL source, the maximal adventitial gradient is under 2 mm$^{-1}$ in over 95% of cases in the healthy configuration (Fig. 12). For $D = 100$ µm$^2$/s ($\mathrm Pe\sim 1$), the value of that maximum is mainly determined by $\textrm{R}_\textrm{d}$, with higher vasa vasorum transwall fluxes increasing the gradient at the EEL and decreasing the concentration in the outer domain. Radially decreasing interstitial convection counteracts solute depletion via microvascular mass fluxes at $D =10$ µm$^2$/s and $D=1$ µm$^2$/s ($\mathrm Pe\sim 10$ and 100, respectively) and results in most concentration profiles being convex in the inner adventitia, with low gradients at the EEL but higher gradients further into the periphery (Table 1).

**Fig. 13:**
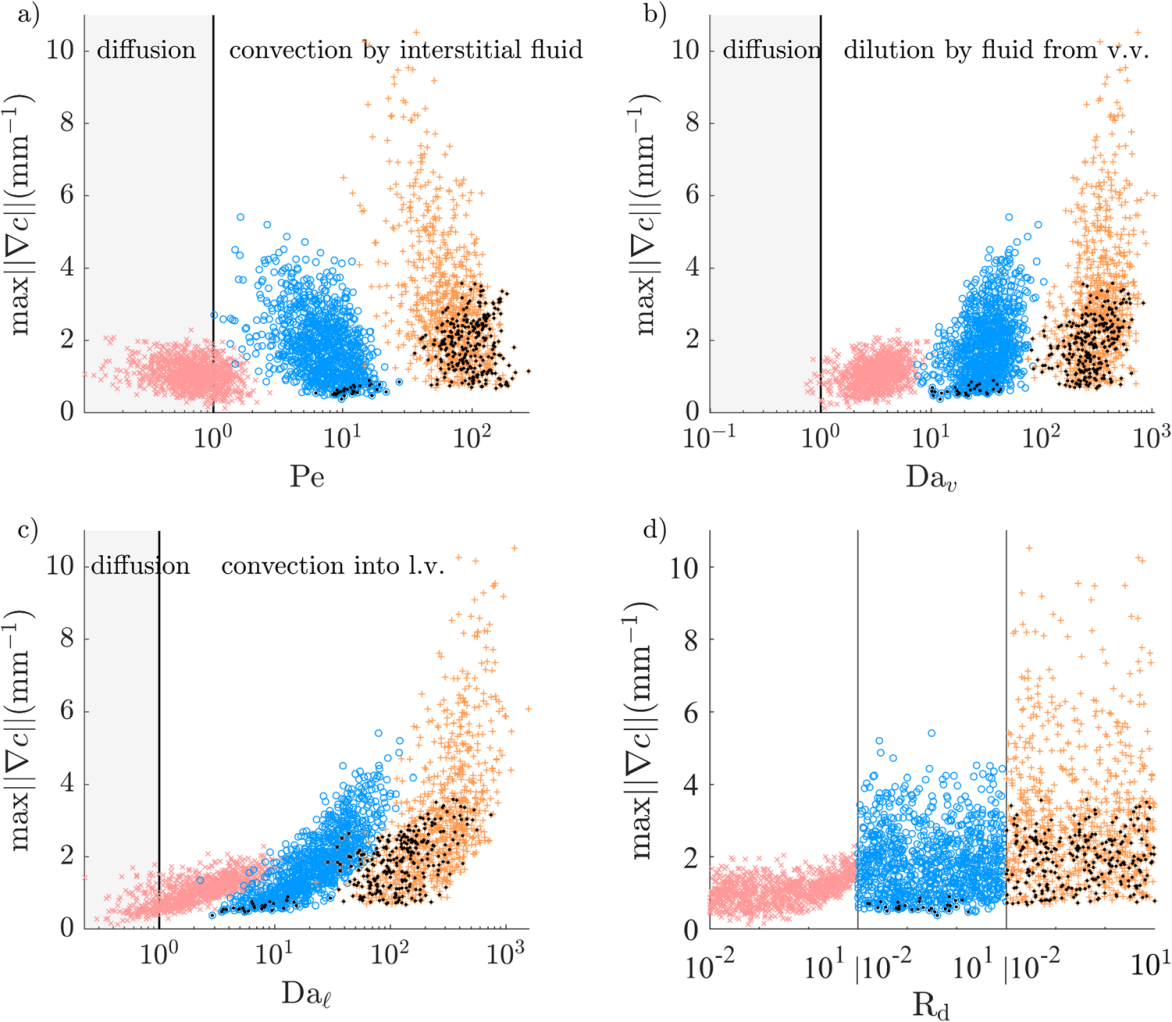
Maximum adventitial gradient (as defined in Fig. 4e) for solutes with an EEL source (atherosclerotic configuration), as a function of the non-dimensional numbers governing Equation (11a). Regions of relative dominance of transport mechanisms separated by grey vertical line. ${\times }\; D=100$ µm$^2$/s, ${\circ }\; D=10$ m$^2$/s, ${+}\; D=1$ m$^2$/s, ${\varvec{\cdot }}$ maximum concentration not at the EEL

The maximal atherosclerotic adventitial gradient is comparable to the healthy one at $D=100$ µm$^2$/s but much larger at $D\le 10$ µm$^2$/s (up to 10 mm$^{-1}$, Fig. 13). The magnitudes of interstitial convection ($\mathrm Pe$) and microvascular fluxes ($\textrm{Da}_v$ and $\textrm{Da}_{\ell }$) are all much larger than that of diffusion and are therefore the main determinants of concentration profiles. High microvascular fluxes result in very large gradients at the EEL, whereas low fluxes allow interstitial convection to enhance transport. The proportions of convex and accumulative profiles are lower than in the healthy configuration because of the slightly reduced interstitial convection and the higher microvascular sinks.

**Table 1:** Proportion of cases with solute source at the EEL presenting convex or accumulative concentration profiles in the adventitia

	Convex (%)			Accumulative (%)		
*D* (µm$^2$/s)	100	10	1	100	10	1
Healthy	10	71	88	0.8	8	32
Atherosclerotic	4	40	54	0.3	3	25

### High atherosclerotic microvascular fluxes can restrict DC–CCL19 transport distance to less than adventitial thickness

**Fig. 14:**
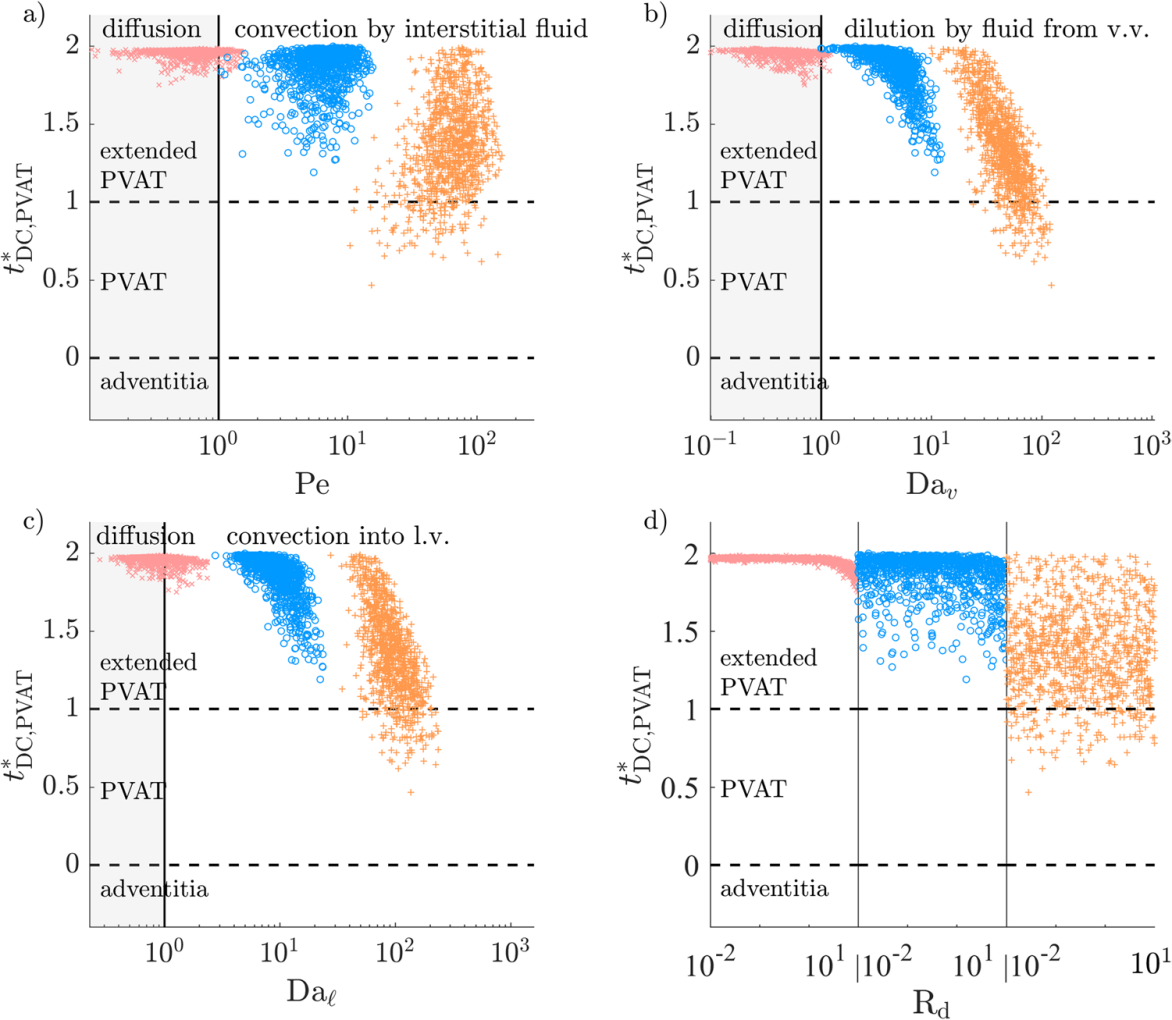
DC–CCL19 transport distance (as defined in Fig. 4f), normalised by PVAT thickness, as a function of the non-dimensional numbers governing Equation (11a) (healthy configuration). Regions of relative dominance of transport mechanisms separated by grey vertical line. ${\times }\; D=100$ µm$^2$/s, ${\circ }\; D=10$ µm$^2$/s, ${+}\; D=1$ µm$^2$/s

For solutes with an EEL source, the DC–CCL19 transport distance is further away than the PVAT thickness in almost all cases of the healthy configuration (Fig. 14). Microvascular sinks reduce that distance when $D \le 10$ µm$^2$/s. In those cases, a large fraction of cells in the extended PVAT would not be able to sense solute gradients that have the DC–CCL19 sensitivity threshold.

**Fig. 15:**
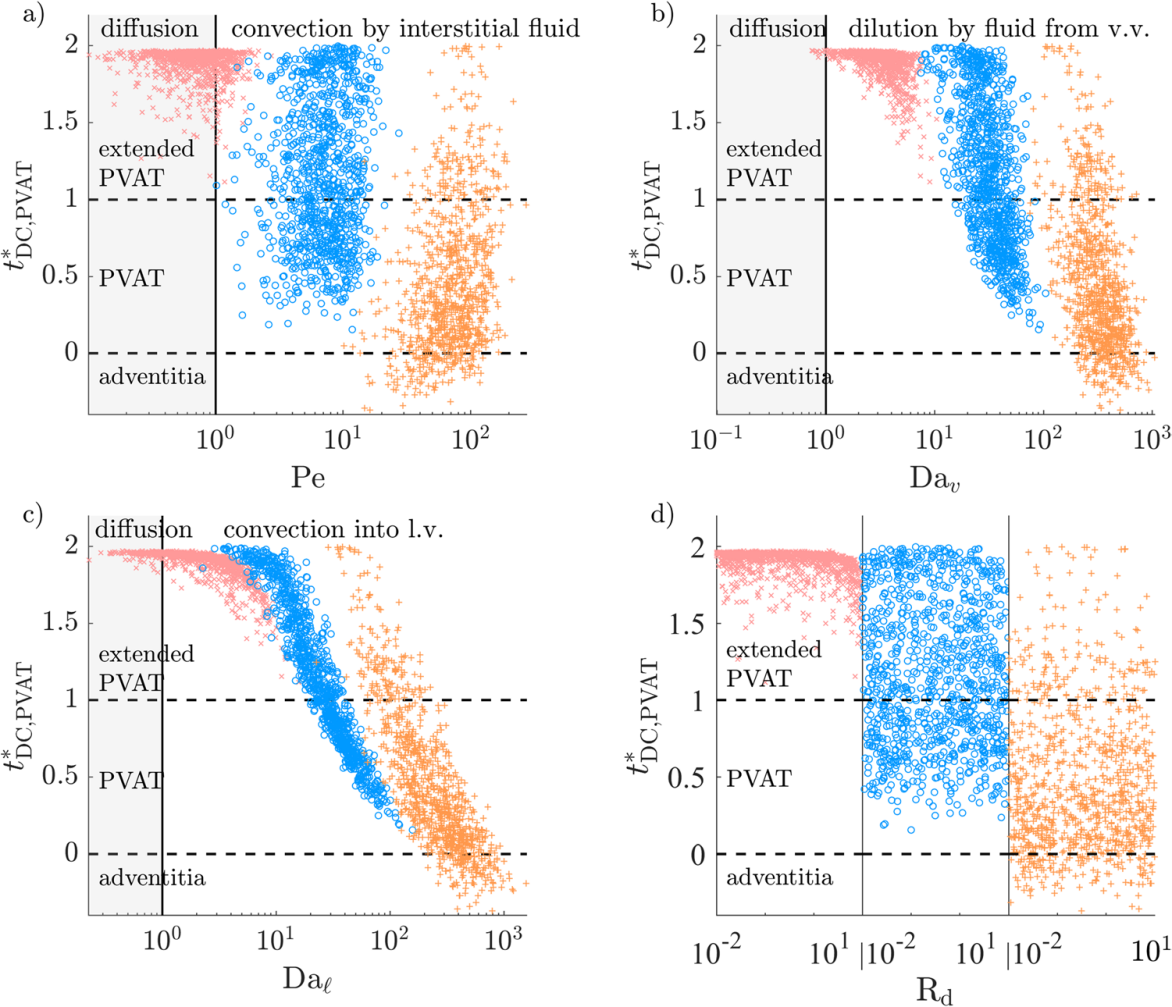
DC–CCL19 transport distance (as defined in Fig. 4f), normalised by PVAT thickness, as a function of the non-dimensional numbers governing Equation (11a) (atherosclerotic configuration). Regions of relative dominance of transport mechanisms separated by grey vertical line. ${\times }\; D=100$ µm$^2$/s, ${\circ }\; D=10$ µm$^2$/s, ${+}\; D=1$ µm$^2$/s

In the atherosclerotic configuration, higher mass exchange fluxes with the microvasculature greatly reduce the DC–CCL19 transport distance when $D\le 10$ µm$^2$/s (Fig. 15). At $D=10$ µm$^2$/s, the distance decreases sharply with $\textrm{Da}_{\ell }$ for $\textrm{Da}_{\ell }>10$ and is further reduced by high $\textrm{Da}_v$. It is in the non-extended PVAT in approximately half of cases. The properties of lymphatic vessels have a higher-order effect than those of vasa vasorum because more lymphatic drainage directly decreases the hydrostatic tissue pressure, which in turn increases the fluid influx from vasa vasorum. At $D=1$ µm$^2$/s, the DC–CCL19 transport distance decreases sharply with $\textrm{Da}_{\ell }$ over its whole range and is under the adventitial thickness in 14% of cases.

## Discussion

### Key results

This model of fluid flow through the artery wall aimed to explore potential effects of ATLOs on fluid and solute transport. Those may be important in atherosclerotic-plaque development and plaque progression towards vulnerability to fracture. A key modelling principle was assuming tissue-wide mass balance between fluid draining from the arterial lumen across its endothelium, fluid flowing across the peripheral vasa vasorum and lymph drained from the wall. Our numerical simulations suggest altered adventitial fluid flow patterns in atherosclerosis. They first suggest elevated interstitial pressure in the adventitia and the PVAT. This is mostly brought about by increases in density and membrane conductivity of vasa vasorum, which decrease trans-endothelial pressure differences. The outer adventitial pressure is calculated to increase from -1±5 mmHg to 4±6 mmHg in atherosclerosis. Second, the combined increases in flow rate from vasa vasorum to adventitia and from adventitia to lymphatics lead to a high dilution of the fluid originating from the arterial lumen. In atherosclerosis, that fluid is calculated to be diluted to 1:10 by plasma from the vasa vasorum before it reaches the outer edge of the adventitia (at 93% of the adventitial thickness on average). This then increases the dilution of disease mediators originating in the lumen or inner layers.

Adventitial solute concentration profiles vary widely depending on the balance between the fluid sources and sinks. They vary from solute accumulation in the inner adventitia to a decrease to near-zero concentration before the adventitia–PVAT boundary. This variation depends largely on fluid balance, which suggests that minor differences in fluid flow could have major implications on the transport of atherogenic or atheroprotective mediators. Those differences directly affect the concentration of immune mediators transported to the wall-draining lymph nodes. Limited or absent lymphangiogenesis and impaired lymphatic function both lead that concentration to increase, which indicates a mechanism by which lymphatic drainage could be atheroprotective.

Altered fluid flow in atherosclerosis affects solute transport from the inner to the outer wall. The presence of a plaque in the intima reportedly increases the inner-layer fluid conductivity (Baldwin et al (1997)), which favours solute transport. However, increased vasa vasorum coverage and conductivity and lymphatic coverage inhibit it. Our model predicts a net inhibition of transport, with greatly reduced transport distances in atherosclerosis. The maximum gradient of a solute of diffusivity 10 µm$^2$/s (assumed to be that of chemokines in a tissue of low porosity like an immune-cell-rich adventitia) increases from 0.9±0.3 mm$^{-1}$ to 1.9±0.9 mm$^{-1}$ in atherosclerosis, which might increase immune-cell retention. Conversely, gradients of VSMC-produced chemokines are in some cases too low to be sensed by cells in the outer PVAT, with the DC–CCL19 sensitivity region shrinking from essentially 100% of the PVAT thickness to 85% on average for a diffusivity of 10 µm$^2$/s.

### Pathophysiological implications

These results provide insight into the potential consequences of changes in tissue morphology and properties that accompany atherosclerosis. ATLOs are known to be central to the immune response to atherosclerosis (Yin et al (2016)). Functional lymphatic drainage in the adventitia has been shown to reduce cholesterol (low-density lipoprotein, LDL) concentration in lesions and contribute to lesion regression (Martel et al (2013)). Lymphatic drainage is also necessary for the cholesterol absorption inhibitor ezetimibe to function (Yeo et al (2020)). Herein, we propose three novel pathways by which changes in tissue morphology and properties may affect disease progression via their effect on adventitial solute concentration. First, increases in vasa vasorum density and vasa vasorum endothelial conductivity reduce solute concentrations via dilution. Second, a combined increase in inner-layer conductivity and impairment of lymphatic drainage favour solute accumulation in the adventitia. Third, immune-cell accumulation in the adventitia reduces adventitial porosity and thus solute diffusivity (e.g. Berson et al (2011)). This leads to higher diffusive fluxes into vasa vasorum relative to those across the tissue and thus greatly reduces solute concentration, particularly for solutes of low molecular weight (high diffusivity). Combined, these three mechanisms lead to a marked reduction in solute transport towards the perivascular space.

The combined effect on disease progression of those mechanisms depends on their relative magnitude, whether they most affect atherogenic or atheroprotective solutes, and the molecular weight of those solutes: the transport of larger solutes would be more severely impaired. In the case of cholesterol, we would expect a reduction in cholesterol concentration in the artery wall. Both LDL (atherogenic) and HDL (atheroprotective) would be affected, but we expect the overall balance to be atheroprotective, as Martel et al (2013) observed when increasing adventitial lymphatic outflow, and because the concentration of LDL (which is larger) would be reduced more than that of HDL. In the case of noradrenaline, which mediates the communication between nerve endings and immune cells in the adventitia (Mohanta et al (2022)), we would also expect an atheroprotective effect. A recent study uncovering the atherogenic role of neuroimmune interfaces reported increases in both axon density, noradrenaline serum levels, and sympathetic nerve activity (Mohanta et al (2022)). This suggests that the noradrenaline release rate of each axon is increased in atherosclerosis. Yet, the increase in noradrenaline serum levels was less pronounced than the axon density increase. This indicates the presence of a mechanism reducing noradrenaline levels that is more active in atherosclerosis. We postulate that to be the increased diffusion of noradrenaline into vasa vasorum. In the case of chemokines produced by VSMCs, their stronger gradients near the EEL favour the attraction and retention of immune cells there, but their weaker gradients in the outer adventitia and the PVAT reduce the attraction of immune cells from those layers. Although this could mean a spatial limitation of immune-cell recruitment, we do not expect that mechanism to contribute to atherosclerosis resistance because the PVAT secretes its own inflammatory mediators that favour the infiltration of immune cells into it (Qi et al (2018)).

### Model refinement

We here suggest avenues for model refinement that could increase its accuracy and refer the reader to the Supplementary Information, Sect. 3, for a detailed discussion of parameter choices.

#### Fluid flow

The inner-layer hydraulic conductivity is likely to depend on plaque type. The studies that reported the inner-layer permeability and thickness (Baldwin et al (1997) and Gradus-Pizlo et al (2003)) do not specify the type of lesion considered, so our study is agnostic to plaque type, but precise modelling of fluid flow across different types of lesions would be a beneficial expansion of the study. Considering the pressure dependency of inner-layer hydraulic conductivity would allow to model the effects of hypertension, but the nature of that dependency is not uniform across studies that report it (Tedgui and Lever (1984); Baldwin et al (1992, 1997)). Measuring or modelling adventitial permeability using fibre matrix theory or image-based simulations would reduce the uncertainty on it and thereby on medial–adventitial pressure. Associating coupled site-specific measurements of vasa vasorum and lymphatic densities (as done for the aorta by Sano et al (2016)) with site-specific modelling of lymphatic drainage (as done for the mesentery by Ikhimwin et al (2020)) could shed light on congruent changes in microvessel density and function and how those regulate interstitial pressure. Finally, site-specific anatomy of microvessel networks will facilitate expanding the model to two and three dimensions and thereby elucidate the distributions of interstitial pressure and solute gradients at a finer scale. Lymphatic vessel distribution indeed varies along the aorta in mice (Yeo et al (2020)), and this ought to locally affect fluid mechanics and solute transport. Expanding the model to more than one dimension would also allow to estimate the small tangential flow and lateral solute gradients resulting from the heterogeneity of arterial lesions and variations in ATLO thickness. Comparing the healthy and atherosclerotic inner-layer velocities and outer adventitial pressures predicted by our model shows that angular heterogeneities are second-order effects compared to the large transwall pressure gradient that is the main driver of interstitial fluid flow.

#### Solute transport

Solute transport is modelled at a steady state, where we assume a separation of time scales between disease-associated changes in cytokine expression by cells of the inner layers (months) and diffusion transport into the peripheral layers (minutes). There are, however, other time scales that would merit attention, e.g. circadian patterns. Their time scale of hours is of the same order of magnitude as diffusion across the arterial periphery and their role in regulating immune processes is receiving increased attention Holtkamp et al (2021); Ince et al (2023). We considered the transport of solutes that do not interact with extracellular matrix and therefore our results concern their transport-limited penetration distance into the arterial periphery, i.e. the maximal distance from the EEL that they can reach. However, many solutes diffusing into the peripheral layers bind to structural proteins and glycosaminoglycans. The chemokines CCL21 and CXCL13, whose increased expression by smooth muscle cells in atherosclerosis is well-documented Grabner et al (2009); Yin et al (2016), notably have a high affinity to heparan sulphate Patel et al (2001). We do not include this in our transport model in the absence of data on glycosaminoglycan expression by stromal cells in the adventitia. Gradients of those chemokines and of other cytokines forming matrix-bound gradients are likely to be of a shorter range, but more stable than the soluble gradients modelled here. We do not include ligand-receptor binding either, which would further shorten the penetration distance into the periphery. Binding kinetics require many parameters to be accurately predicted and ligand-receptor pathways are often multiple (Mamer et al (2020) and Lee et al (2025) illustrate the challenges of predicting ligand binding in the well-studied VEGF/VEGFR pathway). Our model is agnostic to solute type and predicts the transport-limited solute penetration depth. Solute interactions with GAGs and cognate receptors would lower their transport depth and render their inner adventitial gradients higher, but this could be limited or even surmounted by the periodic compression of arterial tissue over a cardiac cycle. Deformation-enhanced transport is a well-studied effect of dynamic loading in many tissues (Shim and Ateshian (2022)) and particularly in articular cartilage, where solute transport can increase up to tenfold at physiologic loading amplitudes and frequencies (Albro et al (2010); DiDomenico et al (2018)). The combination of interstitial fluid flow and ligand-receptor binding kinetics and the consideration of deformation-enhanced transport in arterial tissue would both represent interesting avenues for future work.

#### Perspectives

This exploratory model investigates phenomena that cannot be (or not easily) measured. It indicates phenomena that might be encountered in atherosclerosis and have not yet been elucidated and thereby aims to contribute to new experimental hypotheses. The model and its expansion avenues all aim to respond to the clinical motivation of knowing how far and at which concentration atherosclerosis mediators and drugs are transported into the peripheral wall layers and the wall-draining lymph nodes. Immunotherapies targeting cytokines, and more specifically chemokines, have so far largely failed because of the biological redundancy resulting from the multiplicity of receptor pathways. Models such as this one can serve this aim by offering insight into the concentration of soluble ligands. Their expansion to include spatially resolved production of cytokines and ligand-receptor interactions could contribute to screening therapeutic candidates and refining *in vivo* experiment design.

## Data Availability

The code used to simulate the data presented in this study may be found in a Zenodo repository: https://doi.org/10.5281/zenodo.14833328.

## List of symbols

ATLO: Adventitial tertiary lymphoid organ
PVAT: Perivascular adipose tissue
EEL: External Elastic Lamina
VSMC: Vascular Smooth Muscle Cell
*u*: Interstitial fluid velocity
*p*: Hydrostatic fluid pressure
$\pi$: Osmotic fluid pressure
*c*: Concentration
*k*: Tissue permeability (dimension $L^2$)
*r*: Distance from centre of arterial lumen
*t*: Tissue-layer thickness
$\mu$: Dynamic viscosity of interstitial fluid
*J*: Mass flow rate into tissue per unit volume of tissue
$l_p$: Hydraulic conductivity of vessel wall
*N*: Quantity per unit surface area
$\bar{A}$: Surface area per unit tissue volume
$\sigma$: Osmotic reflection coefficient
$x_\textrm{lu}$: Quantity *x* related to arterial lumen
$x_v$: Quantity *x* related to vasa vasorum
$x_{\ell }$: Quantity *x* related to lymphatic vessels
$c_0$: Concentration at EEL
*D*: Diffusivity in tissue
$\kappa$: Permeability coefficient across vessel wall
$\Phi$: Solute flux into tissue per unit volume tissue
